# Enzyme-Responsive DNA Condensates

**DOI:** 10.1021/jacs.4c08919

**Published:** 2024-11-06

**Authors:** Juliette Bucci, Layla Malouf, Diana A. Tanase, Nada Farag, Jacob R. Lamb, Roger Rubio-Sánchez, Serena Gentile, Erica Del Grosso, Clemens F. Kaminski, Lorenzo Di Michele, Francesco Ricci

**Affiliations:** † Department of Chemical Sciences and Technologies, University of Rome Tor Vergata, Via della Ricerca Scientifica, Rome 00133, Italy; ‡ Department of Chemical Engineering and Biotechnology, University of Cambridge, Philippa Fawcett Drive, Cambridge CB3 0AS, U.K.; § Department of Chemistry, Molecular Sciences Research Hub, Imperial College London, London W12 0BZ, U.K.; ∥ fabriCELL, Molecular Sciences Research Hub, Imperial College London, London W12 0BZ, U.K.

## Abstract

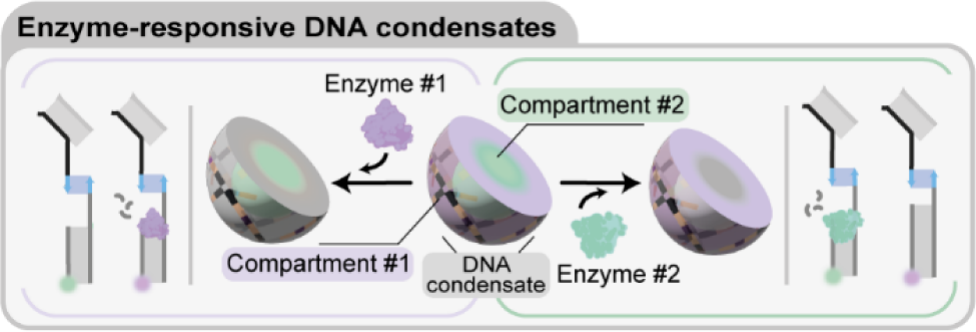

Membrane-less compartments and organelles are widely acknowledged for their role in regulating cellular processes, and there is an urgent need to harness their full potential as both structural and functional elements of synthetic cells. Despite rapid progress, synthetically recapitulating the nonequilibrium, spatially distributed responses of natural membrane-less organelles remains elusive. Here, we demonstrate that the activity of nucleic-acid cleaving enzymes can be localized within DNA-based membrane-less compartments by sequestering the respective DNA or RNA substrates. Reaction-diffusion processes lead to complex nonequilibrium patterns, dependent on enzyme concentration. By arresting similar dynamic patterns, we spatially organize different substrates in concentric subcompartments, which can be then selectively addressed by different enzymes, demonstrating spatial distribution of enzymatic activity. Besides expanding our ability to engineer advanced biomimetic functions in synthetic membrane-less organelles, our results may facilitate the deployment of DNA-based condensates as microbioreactors or platforms for the detection and quantitation of enzymes and nucleic acids.

## Introduction

Living cells are the basic units of life, able to sustain the highly articulate and dynamic functionalities that underpin the emergent behaviors of all living systems. Despite their diversity and complexity, all cells share common features such as the ability to adapt, communicate, process information, grow and divide.^1,2^ Critical to these conserved functionalities are the compartmentalized architectures that separate a cell’s interior from the surrounding environment and maintain its internal heterogeneity. Most cellular pathways are indeed directly controlled by the flow of matter and information across membranes and cell walls, and by the compositional and physical diversity of organelles.^1,3^ For example, compartmentalization is closely connected with enzymatic activity within the cell, which is delicately balanced through multilevel organization and regulation.^4,5^ While cellular compartmentalization often relies on proteolipid membranes, membrane-less compartments emerging from liquid–liquid phase separation or condensation of proteins and nucleic acids are increasingly recognized as ubiquitous and critical in regulating a variety of physiological and pathological processes.^6−8^ Indeed, *membrane-less organelles* found in eukaryotic cells, such as nucleoli, Cajal bodies and P-bodies, are involved in key pathways ranging from transcriptional and post-transcriptional regulation to ribosome biogenesis to RNA degradation.^9−12^

Recent years have seen substantial efforts toward building synthetic cells, artificial devices that display basic features of living cells and show programmable and tunable life-like functionalities, applicable in drug discovery, bioengineering and sensing.^13,14^ Like living cells, synthetic cells require compartments to contain their functional molecular machinery and regulate transport and communication with the outside environment. Membrane-based compartments, created from lipids^15−19^ or polymers^20,21^ constitute a common choice due to their similarity to cellular membranes. Membrane-less enclosures, constructed from synthetic biomolecular condensates, coacervates, or hydrogels, represent valuable alternatives to establish compartmentalization,^22−26^ particularly for their ability to support dynamic behaviors inspired by biological membrane-less organelles.^27^

Owing to the programmability of base pairing, facile synthesis and functionalization, and computational design tools^28−30^ nucleic acid nanotechnology has emerged as a valuable toolkit for engineering both structure and functionalities of synthetic cells.^31−34^ Specifically, synthetic DNA and RNA nanostructures, including branched DNA junctions^35−38^ and single-stranded DNA block copolymers,^39,40^ have been adopted to construct membrane-less compartments with advanced functionalities. These include the ability to capture and release molecular cargoes,^41,42^ host enzymatic reactions,^27,43−45^ interface with live cells,^46,47^ and undergo structural transformations induced by changes in pH,^48,49^ ionic conditions,^50^ or light.^41,51^ Advances have also been made toward establishing physical and chemical heterogeneity within DNA-based membrane-less compartments, for instance by exploiting reaction-diffusion processes^27^ or phase separation.^52−54^ These nonhomogeneous constructs imitate the internal subcompartments observed in several classes of biological membrane-less organelles, including stress granules, nucleoli, L-granules and paraspeckles.^8,9,55−57^

Despite these advances, however, synthetic membrane-less compartments still largely lack the functional complexity of biological ones. Progress remains to be made when implementing dissipative biochemical processes in synthetic condensates, and programming their spatiotemporal distribution within distinct, coexisting subcompartments.

**Figure 1:**
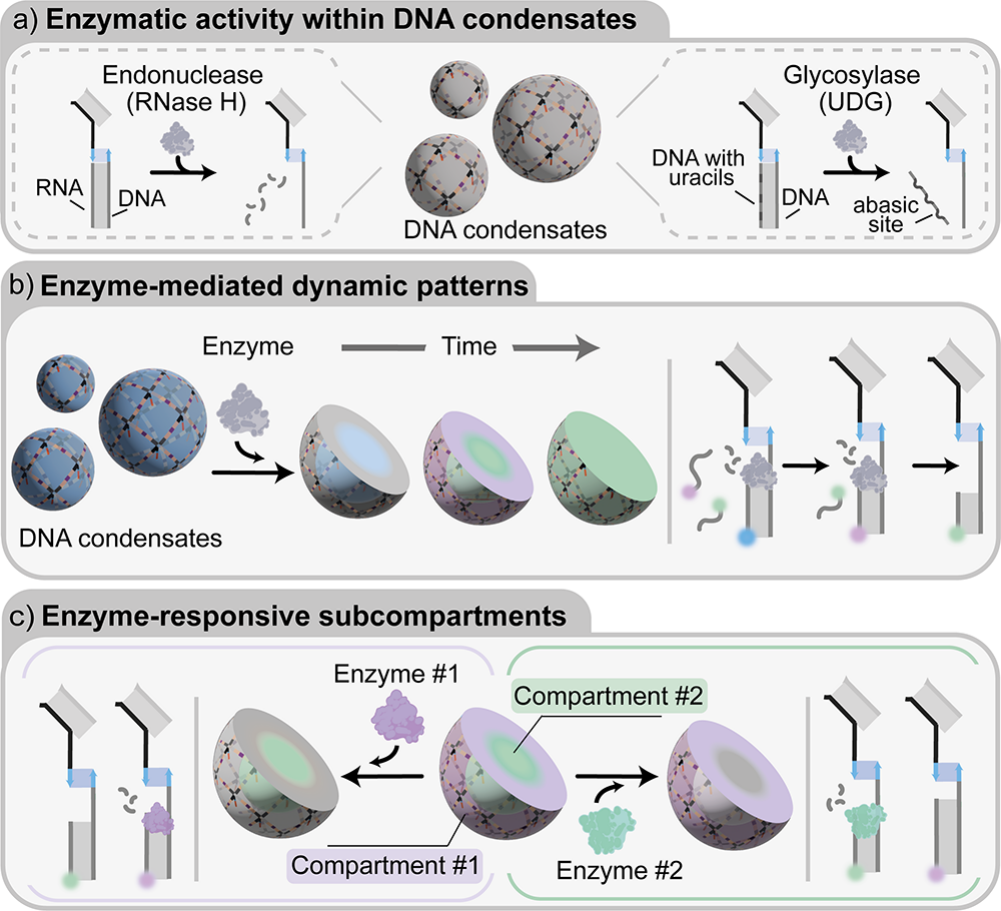
Enzyme-responsive DNA condensates. a) Schematic representations exemplifying the endonuclease and glycosylase activity that can be localized in DNA condensates. b) Schematic representation of enzyme-mediated dynamic patterning of DNA condensates. When added, an enzyme digests nucleic acid substrates bound to the condensates. Digested substrates can be replaced by fresh strands present in solution, producing reaction-diffusion patterns dependent on enzymatic activity, the diffusivity and binding strength of multiple, coexisting substrates. c) Schematic representation of DNA condensates with two concentric subcompartments each containing a different enzymatic substrate, allowing enzymatic reactions to occur only within the predetermined subcompartments.

Here we demonstrate that DNA-based membrane-less synthetic cells can be engineered to localize the activity of different types of enzymes such as an endonuclease and a DNA glycosylase, both targeting specific nucleic-acid substrates (Figure 1a). Exploiting reaction-diffusion mechanisms controlled by the relative size, binding affinity and concentration of the nucleic acid substrates and enzymes, we show that the synthetic cells can be engineered to sustain complex, nonequilibrium patterns that evolve in space and time (Figure 1b). Finally, we use reaction-diffusion processes^27^ to establish static subcompartments in condensates which can be selectively and individually targeted by enzymes, hence demonstrating spatial control over enzymatic activity reminiscent of that observed in natural membrane-less organelles (Figure 1c).^58^

## Result and Discussion

**Figure 2:**
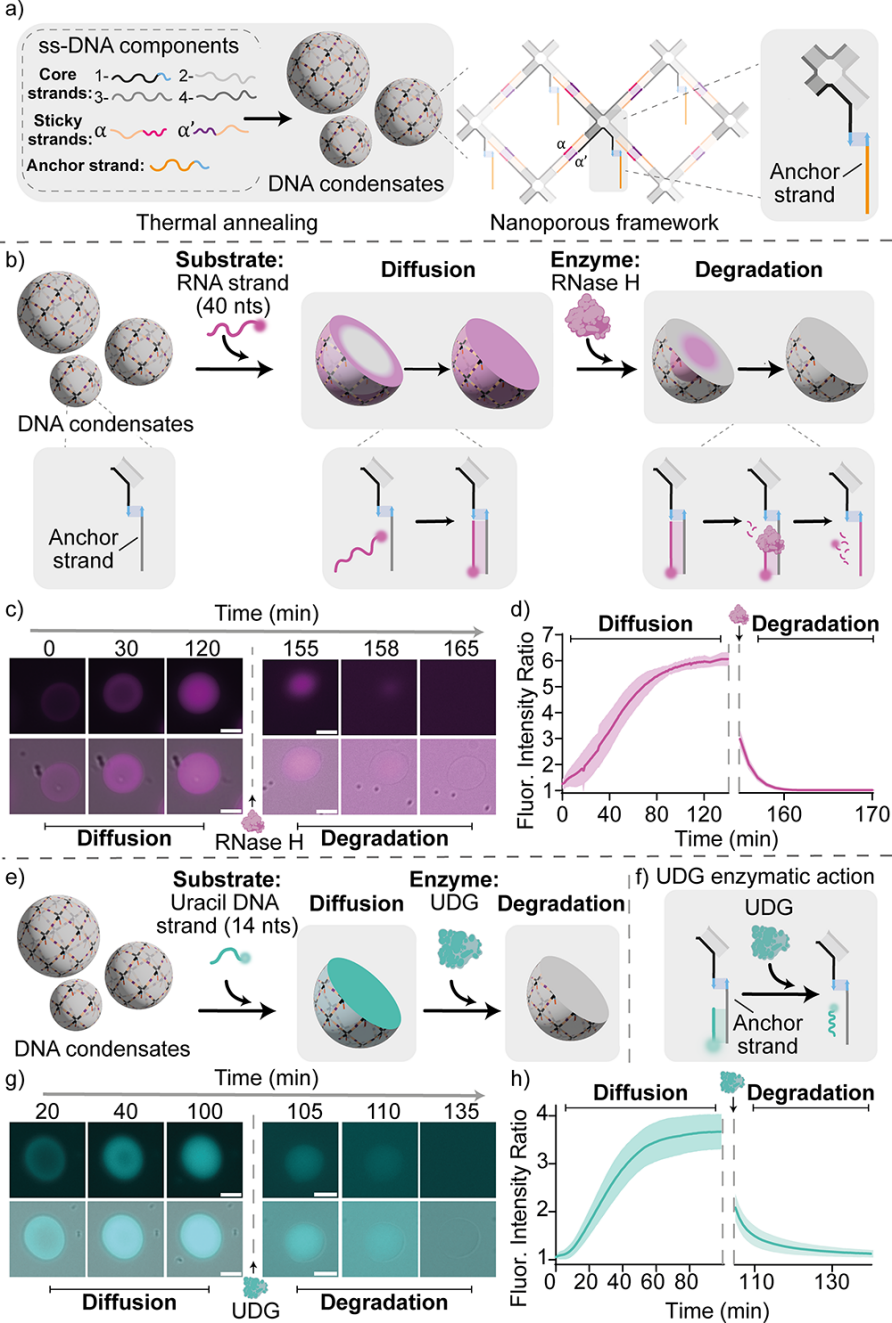
Enzyme-responsive DNA condensates. a) Nanoporous DNA condensates, hosting homogeneously distributed anchor strands, are obtained through slow thermal annealing, from 90 to 20 °C, of the ssDNA components. Full details on nanostructure design and oligonucleotide sequences are reported in the SI (Figure S1). b) Cartoons and reaction schemes illustrating the diffusion and binding of a fluorophore-labeled RNA substrate within a DNA condensate, and its subsequent enzymatic degradation by RNase H. c) Epifluorescence micrographs (top) overlaid with bright-field images (bottom) of the diffusion, binding, and degradation process over time. d) Diffusion/binding and degradation kinetics tracked via the ratio of fluorescent signal samples within the condensates and the surrounding background. Data are shown as mean (solid line) ± standard deviation as obtained analyzing *n* = 352/219 condensates (diffusion stage/degradation stage, respectively) imaged across 3 technical replicates. e, f) Cartoons and reactions schemes illustrating the diffusion and binding of a fluorophore-labeled uracil DNA substrate and its degradation by UDG. g) Epifluorescence micrographs (top) overlaid with bright-field images (bottom) of the diffusion, binding, and degradation process over time. h) Diffusion/binding and degradation kinetics tracked via fluorescence intensity as for panel d. Data are shown as mean (solid line) ± standard deviation as obtained analyzing *n* = 807/155 condensates (diffusion stage/degradation stage, respectively) imaged across 12/3 technical replicates (diffusion stage/degradation stage, respectively). Experiments were performed in Tris HCl 20 mM, EDTA 1 mM, MgCl_2_ 10 mM and 0.05 M NaCl; pH 8.0 at *T* = 30 °C. Sample preparation, annealing process and image analysis details are provided in SI Methods. All scale bars are 10 μm.

We constructed synthetic DNA condensates from the self-assembly of tetravalent DNA nanostructures, termed “nanostars”. As sketched in Figure 2a, individual nanostars fold from four distinct core strands forming a locked four-way DNA junction with 35 base-pair (bp) double-stranded DNA (dsDNA) arms. Each core strand is connected, through hybridization of a 14 nucleotide (nt) domain, to a sticky strand terminating in a 10 nt “sticky end”. Nanostar-nanostar interactions are mediated by hybridization of complementary sticky ends α and *α’*. Each nanostar also displays a 14 nt ssDNA domain (light blue), to which a 54 nt “anchor” strand (blue and orange) can be linked. The anchor strand serves as an addressable binding site for the enzyme-responsive moieties discussed below. Nanostar folding and self-assembly are induced through the one-pot annealing of a stoichiometric mixture of the constituent strands (core strands, anchor strand, sticky strands), from 90 to 20 °C, leading to the formation of spherical condensate droplets with diameters ranging from ∼10 to ∼40 μm (Figures 2a, S1b). The distance between the junctions of two connected nanostars is ∼26 nm (80 bp), ensuring a high porosity of the resulting network, given the rigidity of dsDNA (persistence length ∼50 nm^59^). In particular, we expect the pore size to enable internal diffusion of macromolecular solutes, such as oligonucleotides and proteins.^54,60−62^ Full details on the constituent strand sequences and their assembly into the target structure are provided in Figure S1 and the SI Methods.

Having established nanoporous, addressable DNA condensates, we proceed to demonstrating the localization of enzymatic activity within them. We initially focus on the endonuclease RNase H, an enzyme that degrades RNA only when hybridized to form an RNA/DNA heteroduplex (Figure 2b).^63^ As the target enzymatic substrate for RNase H, we introduce an RNA strand (40 nt, magenta) fully complementary to the anchor strand. The RNA strand is labeled with a fluorophore (Atto 488) so that its localization within the condensates, and subsequent enzymatic degradation, can be monitored through epifluorescence imaging. Upon addition of the RNA strand (200 nM) to a solution containing previously formed DNA condensates (200 nM of the DNA nanostars and anchor strands), we observe an inward-propagating front resulting from the diffusion of the RNA substrates through the DNA condensate, and their subsequent binding to the available DNA anchor strand. The diffusion transient is tracked in Figure 2c,d (left) by monitoring the mean fluorescence intensity within the condensate, determined through image segmentation as outlined in the SI Methods (Section Image analysis). A homogeneous distribution of the RNA substrate through the condensate is achieved within 100 min, as demonstrated in Figure 2c,d (left) and in Movie S1. The spatiotemporal evolution of the substrate distribution can also be visualized with 2D Intensity maps of the time-dependent radial distribution of the fluorescence intensity, *I(r,t)* shown in Figure S2 (see SI Methods, Section Image analysis: Time-dependent radial profiles of fluorescent intensity).

We then added RNase H (50 U/mL, magenta) to the RNA-loaded DNA condensates, observing the propagation of a degradation wave through the condensate as a result of the enzymatic reaction catalyzed by RNase H (Figure 2c,d (right) and Movie S2). The degradation proceeds very rapidly, and the substrate strand is fully degraded within 10 min, as demonstrated in Figures 2d (right), S3c,d and by the *I(r,t)* maps in Figure S3e.

A quick degradation is consistent with the observation that the enzyme has a hydrodynamic diameter of 2.2 nm,^64^ smaller than the estimated mesh size of the DNA networks and enabling rapid diffusion across the condensates. We stress that the enzymatic activity of RNase H does not affect the structure of the DNA condensates, which remain unchanged over the duration of the experiment (Figure S3b–d).

As summarized in Figures S4 and S5, we demonstrated condensate-loading and subsequent digestion for RNA substrates of different length (Movies S3, S4). The three different RNA strands propagate at different rates through the condensate owing to the difference in size and hence diffusion constant.^65^ Degradation of the anchor-bound RNA substrates by RNase H, however, occurs at similar rates, with the 14 nt strand being digested slightly faster owing to its shorter length (Figure S6 and Movies S5, S6).

The same approach for localizing catalytic activity within the condensates can be extended to different enzymes, with the simple expedient of replacing the substrate bound to the anchor strand. To demonstrate this degree of versatility, we used uracil-DNA glycosylase (UDG, hydrodynamic diameter 5.8 nm),^64^ a base-excision repair enzyme that hydrolyses deoxyuridine mutations in DNA strands, leading to the formation of abasic sites.^66^ As the enzymatic substrate, we employed a fluorophore-labeled (Atto 488) 14 nt DNA strand complementary to the anchor strand and containing 4 deoxyuridine mutations (Figure 2e,f). Condensate loading was performed as discussed above, and shown in Figure 2g,h (left) and Movie S7. Upon the addition of UDG (25 U/mL), the enzymatic reaction induces the formation of apurinic sites that destabilize the duplex between the anchor strand and the substrate strand leading to the spontaneous dissociation of the latter. As a result, we observe a dissociation wave that completes within about 20 min (Figures 2g,h right, S7f, and Movie S8) which, as seen for RNase H, does not structurally affect the DNA condensates (Figure S7).

Loading and subsequent digestion of the nucleic-acid substrates occur over different time scales, controlled by the diffusion rates of the macromolecules through the nanoporous condensates, the rates of hybridization to the anchor strands, and the rates of enzymatic digestion. Some of these time scales are controllable by design, e.g., by changing the length of the nucleic-acid substrates or enzyme concentration. The interplay between these time scales can be exploited to establish dynamic compartments, whose composition evolves over time imitating the dynamic character of natural membrane-less organelles.

**Figure 3:**
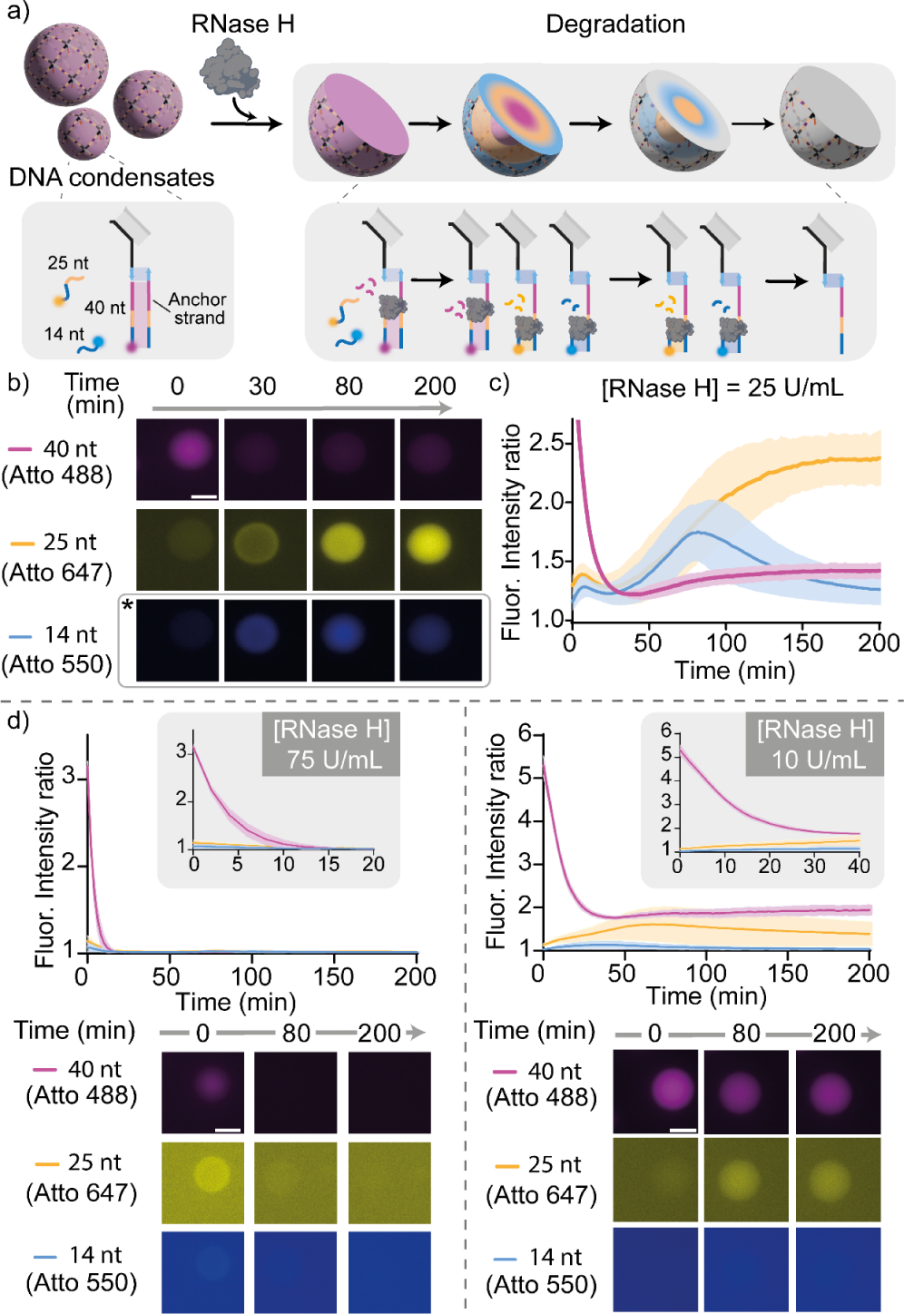
RNase H dynamic compartmentalization. a) Cartoons and reaction schemes illustrating an expected degradation pattern induced by RNase H in the presence of three RNA substrate strands of different lengths (each labeled with a different fluorophore) competing for the anchor strand. b) Epifluorescence micrographs demonstrating the time-evolution of a typical condensate in a sample containing RNase H (25 U/mL) and the three RNA substrate strands (each at 200 nM). For the Atto 550 channel, epifluorescence micrographs are reported with enhanced contrast for better visualization (marked with *). c) Ratio between the fluorescence intensity recorded within the condensate and the surrounding background for the three fluorescent constructs in samples corresponding to the experiment in panel b. Data are shown as mean (solid line) ± standard deviation as obtained analyzing *n* = 195 condensates imaged across 3 technical replicates. d) Top: Ratio between the fluorescence intensity recorded within the condensate and the surrounding background for the three fluorescent constructs. Insets highlight early time scales. Bottom: respective epifluorescence micrographs at different times obtained using a fixed concentration of RNA strands (200 nM) and two different RNase H concentrations: 75 U/ml (left) and 10 U/mL (right). Data are shown as mean (solid line) ± standard deviation as obtained analyzing *n* = 128/222 condensates (RNase H 75 U/mL and 10 U/mL, respectively) imaged across 3 technical replicates. Experiments were performed in Tris HCl 20 mM, EDTA 1 mM, MgCl_2_ 10 mM and 0.05 M NaCl; pH 8.0 at *T* = 30 °C. Sample preparation, annealing process and image analysis details are provided in SI methods. All scale bars are 10 μm.

To demonstrate this functionality, we first exposed the DNA condensates to a mixture of three RNA substrate strands of different length and thus affinity for the anchor strand: 14 nt (Atto 550, blue), 25 nt (Atto 647, yellow), and 40 nt (Atto 488, magenta) (Figure S8). Upon addition of these three strands (200 nM each) to a solution containing the DNA condensates (200 nM of DNA nanostars and of anchor strands) we observe a time-dependent reaction-diffusion pattern, previously reported for DNA strands.^27^ The short (14 nt) strand enters the condensate first owing to faster diffusion, occupying the free anchor strands. The intermediate-length and diffusivity strand (25 nt) follows, displacing the short strand through a toehold-mediated strand displacement reaction.^67,68^ Finally, the longest and slowest-diffusing strand (40 nt) displaces the 25 nt strand, occupying all available anchor strands (Figure S8 b,c and Movie S9). This end point, depicted in Figure 3a (left), corresponds to thermodynamic equilibrium, as the longest 40 nt, substrate strand can bind the anchor strand much more strongly compared to the other substrate strands. We expect the displaced 25 and 14 nt substrate strands to freely diffuse away from the anchor sites, and distribute both outside and inside the condensates at similar concentrations.

Upon adding RNase H, a dynamic reconfiguration is triggered within the condensates. The enzyme, which can only digest RNA when hybridized to DNA, first degrades the 40 nt RNA strands bound to the anchor strand. Removal of the strongest-binding strand allows the shorter strands to repopulate the condensates, as sketched in Figure 3a (right) and experimentally demonstrated in Figure 3b,c. Both the dynamics of the compartment-reconfiguration transient and its end point can be controlled by changing the concentrations of RNase H or of RNA strands. To demonstrate this, we carried out different reactions at a fixed concentration of DNA condensates (200 nM of the DNA nanostars and of anchor strands), in the presence of a mixture of substrate strands (each at 200 nM) and at different concentrations of RNase H (75, 25, and 10 U/mL). At intermediate concentration of RNase H (25 U/mL, Figure 3b,c and Movie S10) we observe the rapid degradation of the longest RNA strand, followed by the two shorter strands rapidly occupying the binding sites made available, with the shortest being slightly faster. A transient configuration in which the two shortest RNA strands are homogeneously distributed through the condensate thus emerges, which rapidly evolves due to the displacement of the shortest strand by the intermediate one (25 nts). Ultimately, the DNA condensates become mainly loaded with the intermediate strand, which remains indefinitely stable due to the progressive loss of activity of the enzyme (Figure 3b,c), in agreement with the time scale of enzymatic activity obtained from bulk fluorimetry experiments (Figure S9, see SI Methods Section Kinetic measurements: Bulk kinetic experiments). After the initial, rapid loss of signal from the longest RNA 40 nt substrate, a slight increase in fluorescence is reported at later times. This increase is ascribed to a slight excess of the 40 nt substrates, which can thus displace some of the 25 nt strands and bind to the anchors (see SI Methods Section DNA condensate assembly). The dynamic compartment-reconfiguration triggered by RNase H can also be visualized with the *I(r,t)* maps, shown in Figure S10. Using a higher (75 U/mL) or lower (10 U/mL) concentration of RNase H results in distinctively different diffusion-degradation pathways, as demonstrated in Figure 3d by sampling the fluorescent signal from the three RNA substrates at three different times (0, 80, and 200 min). With [RNase H] = 75 U/mL, after the longest RNA is removed, the two others are degraded by the enzyme as soon as they bind the free anchor strands, and neither persists indefinitely. In turn, with 10 U/mL of RNase H, the longest RNA strand is only partially removed before loss of enzymatic activity, triggering occupation of some of the binding sites by the intermediate strand.

**Figure 4:**
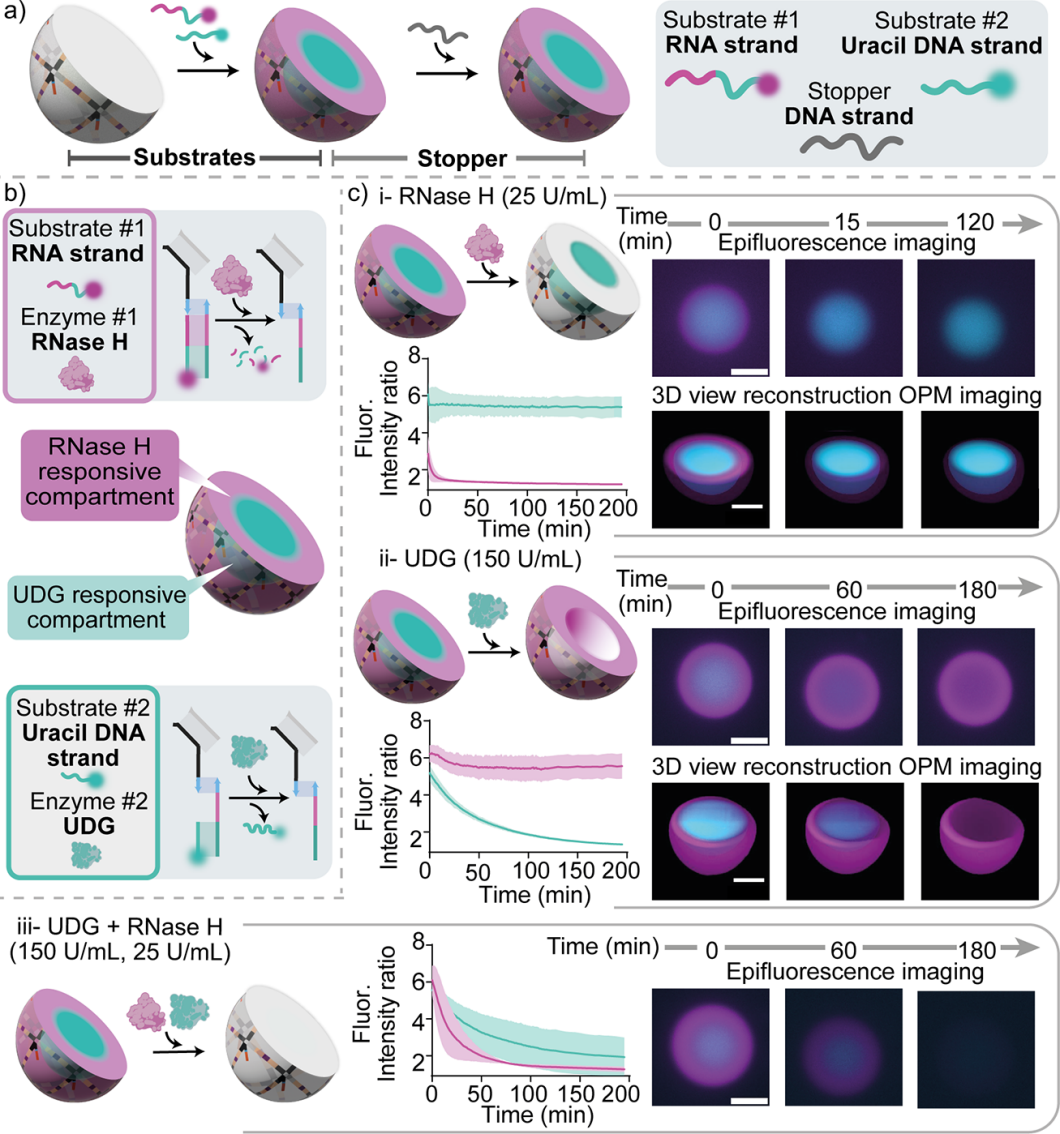
RNase H and UDG responsive compartments in DNA condensates. a) Cartoon illustrating the formation of membrane-less compartments in DNA condensates. The two substrate strands, namely the uracil DNA strand (25 nt, Atto 488 labeled, cyan) and the RNA strand (40nt, Atto 647 labeled, magenta), establish a core–shell pattern within the DNA condensates through a reaction-diffusion process. Adding an excess of the stopper strand arrests pattern propagation by sequestering unbound substrate strands, resulting in the formation of two stable, concentric membrane-less compartments enriched in the two different substrates. b) Cartoons illustrating the two responsive compartments in a DNA condensate: an external one (magenta) hosting the substrate of RNase H and an internal one (cyan) containing the substrate of UDG. c) Epifluorescence micrographs (top right), 3D reconstructions obtained from Oblique Plane Microscopy (bottom right) and fluorescence intensity kinetics (left, as sampled with epifluorescence) demonstrating localized, orthogonal and specific enzymatic activity within the condensates, by adding RNase H only (i), UDG only (ii) or both enzymes (iii). RNase H and UDG concentrations were fixed at 25 U/mL and 150 U/mL, respectively. RNA and uracil DNA substrates were fluorescently labeled with Atto 647 and Atto 488, respectively. Data are shown as mean (solid line) ± standard deviation as obtained analyzing *n* = 115/93/91 condensates (respectively i, ii, and iii) images across 3 technical replicates. Note that the subcompartments established within the condensates do not change morphology over time, confirming that the condensates are in a solid phase and internal diffusion of the DNA nanostars (and anchor strands connected to them) does not occur over relevant experimental time scales. Experiments were performed in Tris HCl 20 mM, EDTA 1 mM, MgCl_2_ 10 mM and 0.05 M NaCl; pH 8.0 at *T* = 30 °C. Sample preparation, annealing process and image analysis details are provided in the SI Methods. All scale bars are 10 μm.

Having demonstrated the use of enzymatic reactions to program time-dependent responses, we proceed to show that activity can be localized in distinct, addressable subcompartments, thus achieving spatial organization of functionalities akin to that observed in biological condensates. As substrates for these reactions, we used two different nucleic acid strands: an RNA strand as the substrate of RNase H and an uracil-containing DNA strand as the substrate of UDG. Also, in this case the two substrate strands are labeled with two different fluorophores, so that their diffusion (and subsequent enzymatic removal) can be easily followed through fluorescence imaging. We proceeded to precisely localize these two nucleic acid substrates in distinct, concentric regions within condensates by employing a technique already optimized in a previous contribution and summarized in Figure S11.^27^ Specifically, adding the two strands in a solution containing the condensates results in a time-dependent reaction diffusion-pattern whereby the shorter (25 nt), faster-diffusing UDG substrate (Atto 488, cyan) initially diffuses and binds within the condensate, and is later displaced by the longer (40 nt), slower diffusing RNase H substrate (Atto 647, magenta) from the outside of the condensate inward. A transient pattern is thus established, with the UDG substrate localized in the condensate’s core and the RNase H substrate in its outer shell. To arrest the pattern in this configuration, it is sufficient to add a large excess of a stopper strand (5 μM, 40 nt) with the same sequence of the binding site on the anchor strand, which captures all the free substrate strands present in solution. Once excess substrates are sequestered, the strand displacement reactions leading to pattern propagation are no longer possible, freezing the core–shell pattern in place and resulting in the formation of two concentric membrane-less compartments within the DNA condensates (Figures S11, 4a, Movies S11 and S12). We then proceeded to expose the patterned DNA condensates to one of, or both, the relevant enzymes, and to characterize the localized enzymatic activity with video microscopy and image analysis (Figure 4b).

We observe that enzymatic actions are mutually orthogonal and only occur within the target subcompartments (Figure 4c). When only RNase H is added, enzymatic digestion is localized only in the external region (magenta) and the RNA strand is removed in about 15 min (Figure 4c-i top, Movie S13). If only UDG is added, the enzymatic activity is instead localized solely in the inner compartment, resulting in the removal of the uracil strand (Figure 4c-ii middle, Movie S14). In both cases, after the removal of the desired substrate strand, no free substrate strands are present and able to bind to the newly available anchor strands, given that all free substrate strands were previously captured by the stopper strand (see SI Methods and Figures S11, S12). Volumetric reconstructions obtained through time-lapse Oblique Plane Microscopy (OPM) confirm the intended three-dimensional morphology of the patterned condensates, and the selective targeting of the outer shell and inner core when the condensates are exposed to RNase H or UDG, respectively (3D views in Figure 4c-i and ii and Movies S15 and S16). OPM is a light sheet-based imaging modality that employs a single objective at the sample,^69^ allowing for the high spatial and temporal resolution afforded by light sheet imaging to be utilized with standard sample mounting technologies. Consequently, OPM is capable of imaging entire condensates at a temporal resolution unachievable by other 3D imaging technologies, such as laser scanning confocal microscopy. This provides an enhanced ability to study the three-dimensional dynamic response of the condensates upon the addition of the enzymes. If condensates are exposed to both UDG and RNase H, both substrate strands are removed (Figures 4c-iii bottom, S12 and Movie S17). The time-dependent radial profile of the fluorescence intensity, *I(r,t),* confirms the orthogonal and spatially targeted enzymatic activity within the core–shell patterned DNA condensates (Figure S13). We point out that the difference in degradation time scales between the two substrates is likely due to the different mechanism of activity of the two enzymes.

## Conclusions

Here we demonstrated that membrane-less, DNA-based condensates (synthetic cells), self-assembled from a small number of DNA and RNA oligonucleotides, can be engineered to sustain complex spatiotemporal patterns sustained by enzymatic reactions. The activity of DNA-repair enzymes RNase H and UDG is localized within the synthetic cells by hybridizing their target nucleic-acid substrates to dedicated binding sites. The nanoporous nature of the condensates facilitates the diffusion of substrates, enzymes and products, enabling the emergence of complex nonequilibrium patterns that evolve in space and time, regulated by the relative size of the substrates, their affinity for the condensates and the concentration of the enzymes. Reaction-diffusion patterns can be arrested to precisely localize substrates in subcompartments within the condensates, which can then be selectively targeted by the corresponding enzymes.

The nonequilibrium pattern formation and the spatial control of enzymatic activity are key characteristics of biological membrane-less organelles, and the ability to recapitulate them in artificial analogues could be highly valuable for the deployment of synthetic DNA condensates in bottom-up synthetic biology. For instance, one could consider exploiting enzyme-activity localization to optimize enzymatic cascades hosted within synthetic cells, applicable to biomanufacturing and biosensing.^3,70−72^ Similar strategies demonstrated here to achieve enzyme-responsive patterns in DNA condensates could also be employed for controlling the patterns in other DNA self-assembled materials and thus lead to new ways to program materials at the nanoscale using enzymatic reactions.

In addition to expanding the temporal control over enzymatic activity with nucleic acids,^73−75^ the enzyme-dependent spatiotemporal patterns could be valuable as readouts for synthetic-cell based assays aimed at detecting substrates, enzymes and quantifying enzymatic activity, as relevant for diagnosing conditions characterized by dysregulation of enzymes or circulating nucleic acids.^76−78^

## Supporting Information Available

Oligonucleotide sequences used, protocols, experimental methods, supplementary figures, image analysis, and supplementary videos description (PDF)
Movie S1: Diffusion of substrate RNA strand 40 nt (MP4)
Movie S2: Degradation of substrate RNA strand 40 nt in the presence of 50 U/mL of RNase H (MP4)
Movie S3: Diffusion of substrate RNA strand 14 nt (MP4)
Movie S4: Diffusion of substrate RNA strand 25 nt (MP4)
Movie S5: Degradation of substrate RNA strand 14 nt in the presence of 50 U/mL of RNase H(MP4)
Movie S6: Degradation of substrate RNA strand 25 nt in the presence of 50 U/mL of RNase H (MP4)
Movie S7: Diffusion of substrate Uracil strand 14 nt (MP4)
Movie S8: Degradation of substrate Uracil strand 14 nt of 25 U/mL of UDG (MP4)
Movie S9: Simultaneous diffusion of three RNA substrates with different lengths: 40 nt, 25 nt and 14 nt (MP4)
Movie S10: Dynamic pattern compartmentalization with RNase H 25 U/mL (MP4)
Movie S11: Formation of DNA condensates with core–shell compartments: Diffusion (MP4)
Movie S12: Formation of DNA condensates with core–shell compartments: Stopper (MP4)
Movie S13: DNA condensates with core–shell compartments exposed to RNase H (MP4)
Movie S14: DNA condensates with core–shell compartments exposed to UDG (MP4)
Movie S15: 3D view reconstruction of DNA condensates with core–shell compartments exposed to RNase H (MP4)
Movie S16: 3D view reconstruction of DNA condensates with core–shell compartments exposed to UDG (MP4)
Movie S17: DNA condensates with core–shell compartments exposed to both RNase H and UDG (MP4)
